# Supplementary material for: Nutrition Related Stress Factors Reduce the Transfer of Extended-Spectrum Beta-Lactamase Resistance Genes between an Escherichia coli Donor and a Salmonella Typhimurium Recipient In Vitro
Source: Biomolecules. 2019 Jul 31;9(8):324. doi: 10.3390/biom9080324 (PMC6724058; doi:10.3390/biom9080324)
Supplement: Supplementary file 1 [file biomolecules-09-00324-s001.zip › Supplementary Data_biomol.docx]

# Supplementary Data

[Figure S1: Bacterial concentration at different pH-values. 3](#_Toc14784502)

[Figure S2: Bacterial concentration at different osmolalities.. 3](#_Toc14784503)

[Figure S3: Bacterial concentration at different levels of cefotaxime (CTX).](#_Toc14784504)

[Figure S4: Bacterial concentration at different different levels of sulfamethoxazole/ trimethoprim (SXT).. 4](#_Toc14784505)

[Figure S5: Bacterial concentration at different levels of nitrofurantoin (F). 5](#_Toc14784506)

[Figure S6: Bacterial concentration at different levels of copper.. 5](#_Toc14784507)

[Figure S7: Bacterial concentration at different levels of zinc.. 6](#_Toc14784508)

[Figure S8: Bacterial concentration at different concentrations of acetate. 6](#_Toc14784509)

[Figure S9: Bacterial concentration at different concentrations of D/L-lactate. 7](#_Toc14784510)

[Figure S10: Bacterial concentration at different concentrations of proprionate. 7](#_Toc14784511)

[Figure S11: Bacterial concentration at different concentrations of n-butyrate. 8](#_Toc14784512)

# Nutrition related stress factors reduce the transfer of extended-spectrum beta-lactamase resistance genes between an *Escherichia coli* donor and a *Salmonella* Typhimurium recipient *in vitro*

Eva-Maria Saliu*, Wilfried Vahjen, Marita Eitinger and Jürgen Zentek

Freie Universität Berlin, Institute of Animal Nutrition, Königin-Luise-Str. 49, 14195 Berlin, Germany

Correspondence: Eva-Maria Saliu [Eva-Maria.Saliu@fu-berlin.de](mailto:Eva-Maria.Saliu@fu-berlin.de)


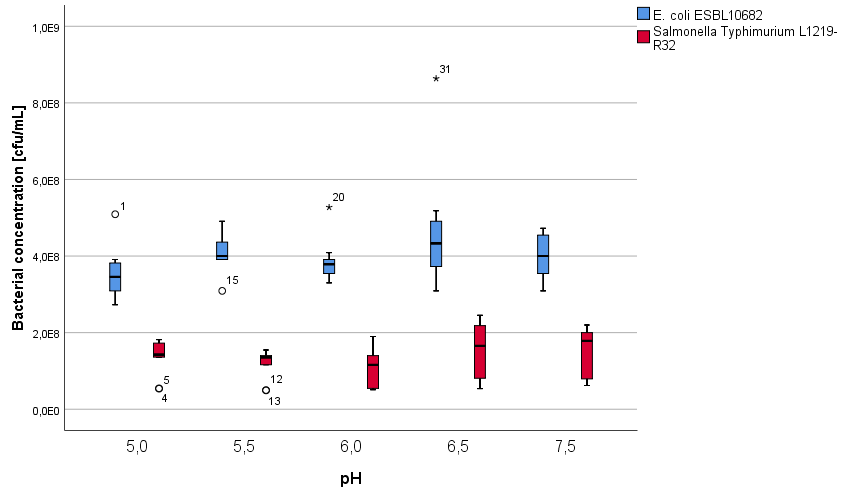


Figure S1: Bacterial concentration after 4 hours co-incubation of E. coli ESBL10682 and Salmonella Typhimurium L1219-R32 in Mueller Hinton 2 Broth with different pH-values. The boxplot displays median, interquartiles, and 95th and 5th percentile as well as outliers (○) and extreme outliers (*).


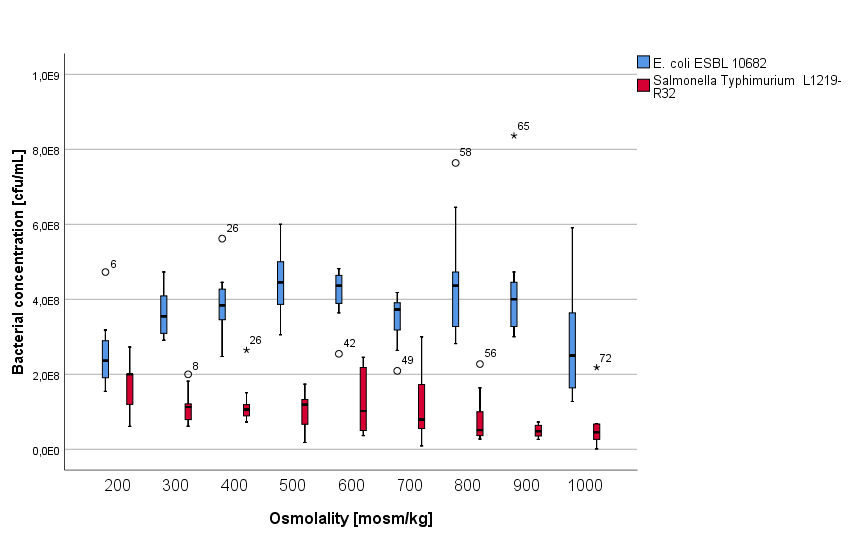


Figure S2: Bacterial concentration after 4 hours co-incubation of E. coli ESBL10682 and Salmonella Typhimurium L1219-R32 in Mueller Hinton 2 Broth with different osmolalities. The boxplot displays median, interquartiles, and 95th and 5th percentile as well as outliers (○) and extreme outliers (*).


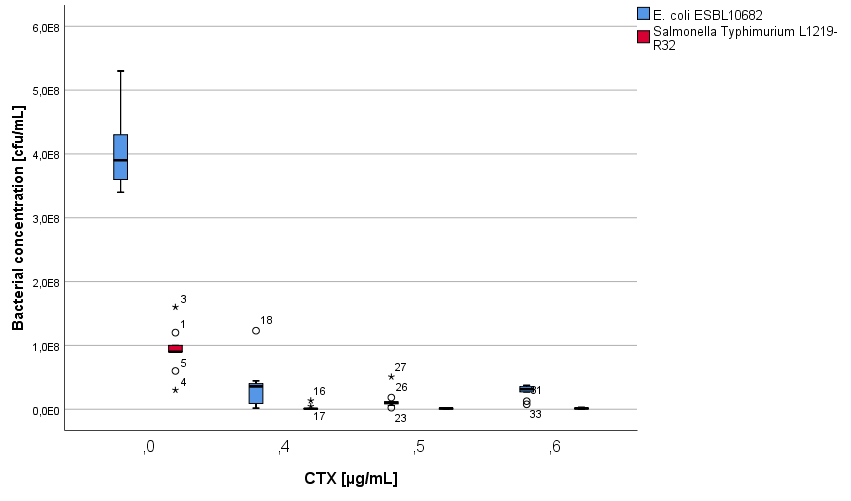


Figure S3: Bacterial concentration after 4 hours co-incubation of E. coli ESBL10682 and Salmonella Typhimurium L1219-R32 in Mueller Hinton 2 Broth with different levels of cefotaxime (CTX). The boxplot displays median, interquartiles, and 95th and 5th percentile as well as outliers (○) and extreme outliers (*).


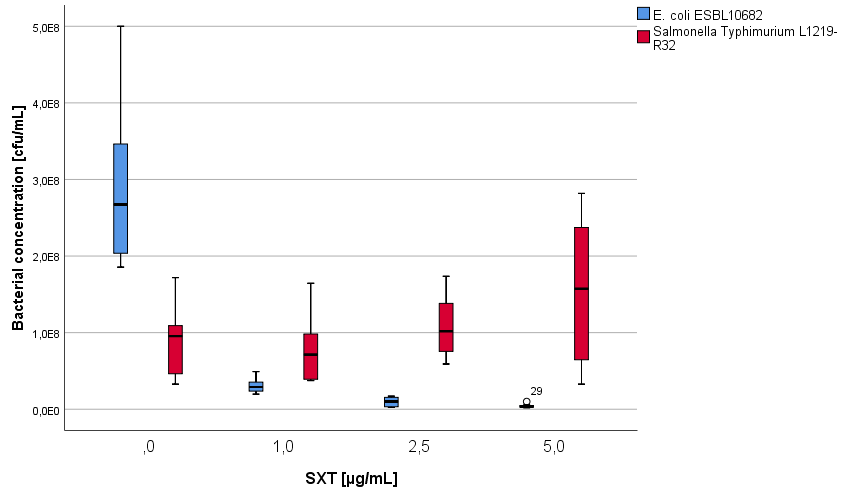


Figure S4: Bacterial concentration after 4 hours co-incubation of E. coli ESBL10682 and Salmonella Typhimurium L1219-R32 in Mueller Hinton 2 Broth with different levels of sulfamethoxazole/trimethoprim (SXT). The boxplot displays median, interquartiles, and 95th and 5th percentile as well as outliers (○) and extreme outliers (*).


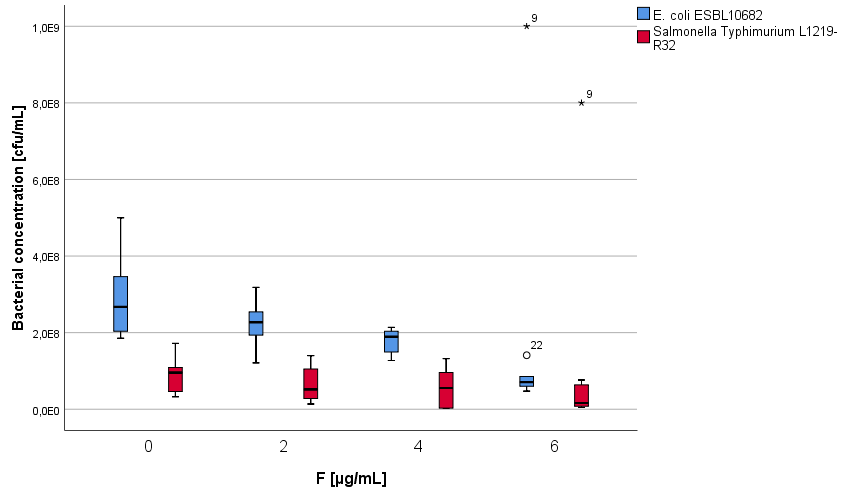


Figure S5: Bacterial concentration after 4 hours co-incubation of E. coli ESBL10682 and Salmonella Typhimurium L1219-R32 in Mueller Hinton 2 Broth with different levels of nitrofurantoin (F). The boxplot displays median, interquartiles, and 95th and 5th percentile as well as outliers (○) and extreme outliers (*).


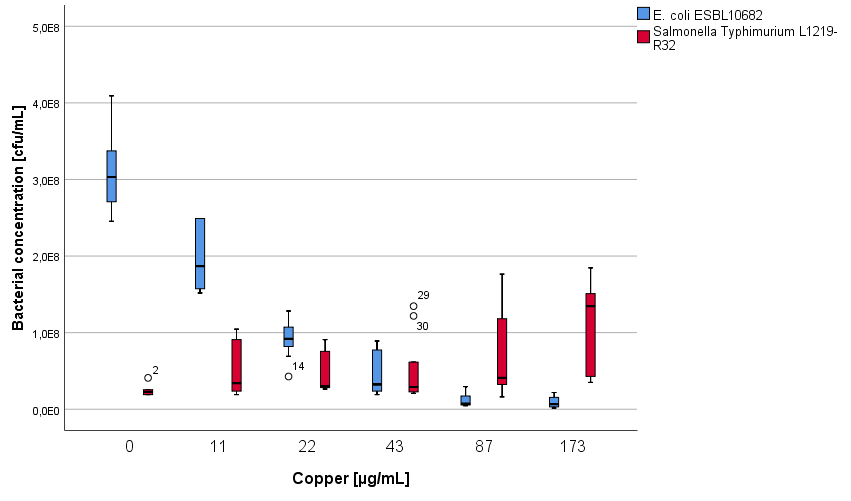


Figure S6: Bacterial concentration after 4 hours co-incubation of E. coli ESBL10682 and Salmonella Typhimurium L1219-R32 in Mueller Hinton 2 Broth with different levels of copper. The boxplot displays median, interquartiles, and 95th and 5th percentile as well as outliers (○) and extreme outliers (*).


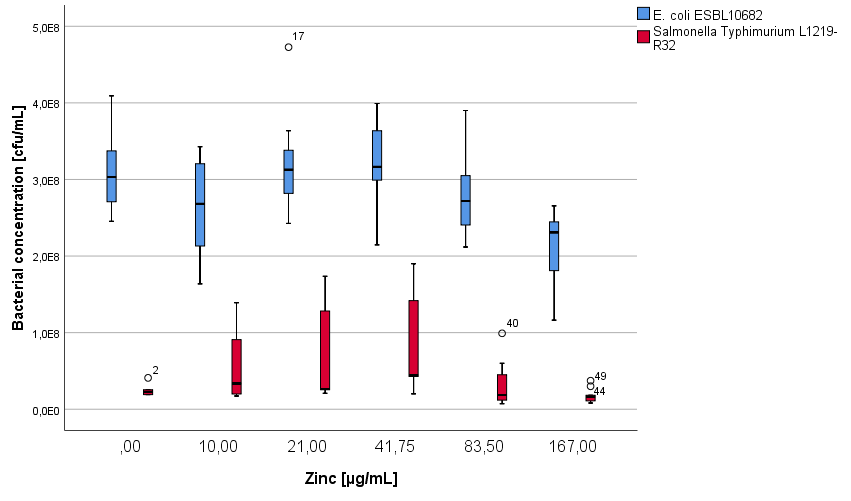


Figure S7: Bacterial concentration after 4 hours co-incubation of E. coli ESBL10682 and Salmonella Typhimurium L1219-R32 in Mueller Hinton 2 Broth with different levels of zinc. The boxplot displays median, interquartiles, and 95th and 5th percentile as well as outliers (○) and extreme outliers (*).


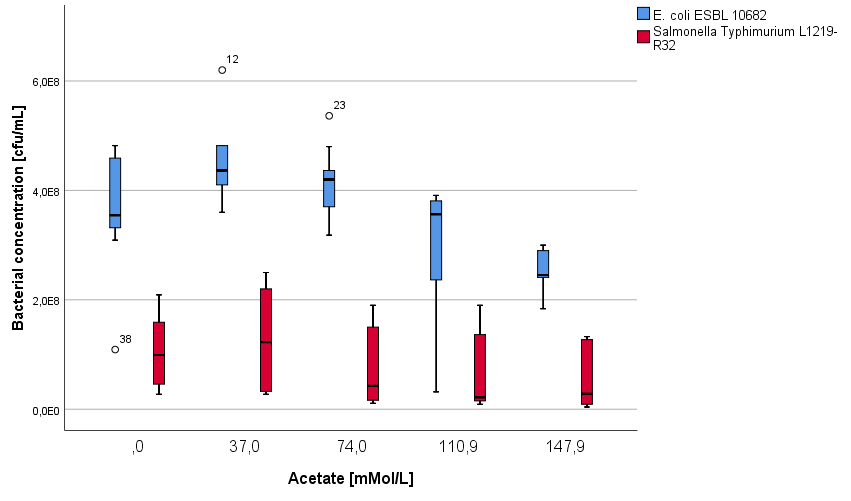


Figure S8: Bacterial concentration after 4 hours co-incubation of E. coli ESBL10682 and Salmonella Typhimurium L1219-R32 in Mueller Hinton 2 Broth with different concentrations of acetate. The boxplot displays median, interquartiles, and 95th and 5th percentile as well as outliers (○) and extreme outliers (*).


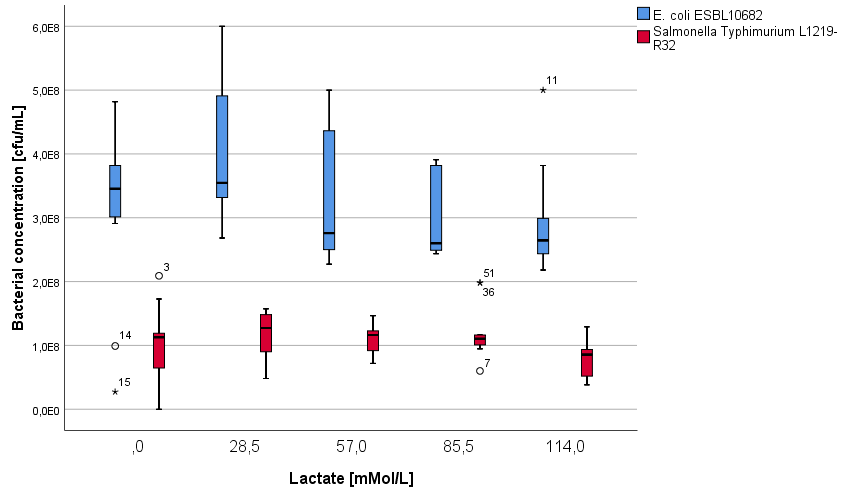


Figure S9: Bacterial concentration after 4 hours co-incubation of E. coli ESBL10682 and Salmonella Typhimurium L1219-R32 in Mueller Hinton 2 Broth with different concentrations of D/L-lactate. The boxplot displays median, interquartiles, and 95th and 5th percentile as well as outliers (○) and extreme outliers (*).


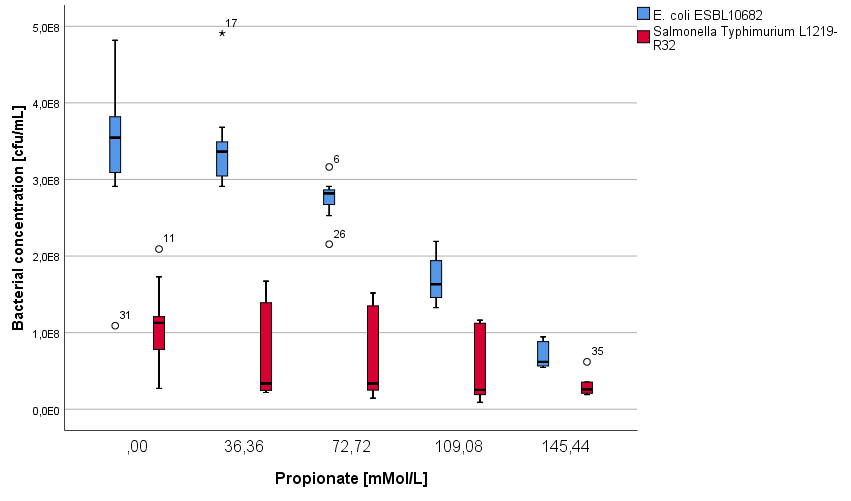


Figure S10: Bacterial concentration after 4 hours co-incubation of E. coli ESBL10682 and Salmonella Typhimurium L1219-R32 in Mueller Hinton 2 Broth with different concentrations of proprionate. The boxplot displays median, interquartiles, and 95th and 5th percentile as well as outliers (○) and extreme outliers (*).


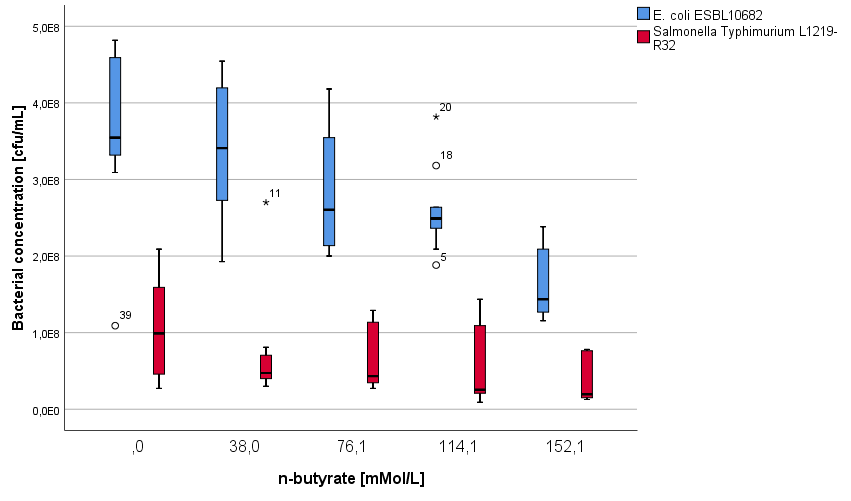


Figure S11: Bacterial concentration after 4 hours co-incubation of E. coli ESBL10682 and Salmonella Typhimurium L1219-R32 in Mueller Hinton 2 Broth with different concentrations of n-butyrate. The boxplot displays median, interquartiles, and 95th and 5th percentile as well as outliers (○) and extreme outliers (*).
